# Monthly Report of the New York Dispensaries

**Published:** 1856-10

**Authors:** 


*Monthly Report of the New York Dispensaries.*—Aggregate number to whom medical services and medicine were rendered gratuitously during the month of July: Males, 3,212; females, 5,025. Born in the United States, 2,835; born in foreign countries, 5,402. Sent to the hospital, 310, died, 88. The principal causes of death were—cholera infantum, consumption and marasmus. The prevailing diseases generally affected the digestive systems: among the most important were—cholera infantum, diarrhœa and marasmus.—*N. Y. Times.*

